# Exploration of Mental Health Elements in Chemsex Behavior: Interventions, Influencing Factors, and Attitudes towards Risk and Harm Reduction in Spain

**DOI:** 10.3390/nursrep14030172

**Published:** 2024-09-06

**Authors:** Pablo Del Pozo-Herce, Alberto Tovar-Reinoso, Antonio Martínez-Sabater, Elena Chover-Sierra, Teresa Sufrate-Sorzano, Carlos Saus-Ortega, Javier Curto-Ramos, José Manuel Padilla-Brito, Carlos González-Navajas, Enrique Baca-García, Raúl Juárez-Vela, Eva García-Carpintero Blas

**Affiliations:** 1 Department of Psychiatry, Fundación Jiménez Díaz University Hospital, 28040 Madrid, Spain; pablo.pozo@quironsalud.es (P.D.P.-H.); ebaca@quironsalud.es (E.B.-G.); 2 Instituto de Investigación Sanitaria de la Fundación Jiménez Díaz, 28040 Madrid, Spain; 3 UNIE Universidad, 28040 Madrid, Spain; 4 Nursing Care and Education Research Group (GRIECE), GIUV2019-456, Nursing Department, Universitat de Valencia, 46010 Valencia, Spain; antonio.martinez-sabater@uv.es; 5 Care Research Group (INCLIVA), Hospital Clínico Universitario de Valencia, 46010 Valencia, Spain; elena.chover@uv.es; 6 Internal Medicine, Consorci Hospital General Universitari de Valencia, 46014 Valencia, Spain; 7 Department of Nursing, Faculty of Health Sciences, Research Group in Care GRUPAC, University of La Rioja, 26006 Logroño, Spain; raul.juarez@unirioja.es; 8 Nursing School La Fe, Adscript Centre, University of Valencia, 46026 Valencia, Spain; saus_car@gva.es; 9 Research Group GREIACC, Health Research Institute La Fe, 46016 Valencia, Spain; 10 Department of Psychiatry, Clinical Psychology and Mental Health, La Paz University Hospital, 28046 Madrid, Spain; javier.curto@salud.madrid.org; 11 Department of Pneumology, Hospital Clinico San Carlos, 28040 Madrid, Spain; josemanuel.padilla@salud.madrid.org; 12 Child and Adolescent Psychiatry and Psychology Service, Child Hospital Niño Jesus, 28009 Madrid, Spain; pretiums@gmail.com; 13 NBC Group, Health Department, School of Life and Nature Sciences, Nebrija University, 28240 Madrid, Spain; egarcibl@nebrija.es

**Keywords:** chemsex, men who have sex with men, harm reduction, qualitative research, sexual behavior

## Abstract

In recent years, there has been an increase in the practice of chemsex, which is becoming an increasingly serious public health problem. The complex interaction between chemsex and mental health underscores the need to understand the psychological, social, and environmental factors that influence this practice. Methods: A qualitative descriptive phenomenological study was conducted in the community of Madrid, Spain to explore the depth of the chemsex phenomenon through a thematic analysis. Between April and June 2024, interviews were conducted with 15 MSM (gay, bisexual, and other men who have sex with men) who participate in these practices, using purposive and snowball sampling. Results: three main themes were identified, namely, (T1) contextualization of the practice, (T2) factors associated with chemsex, and (T3) strategies and interventions for risk and harm reduction. Conclusion: Chemsex is a phenomenon that combines sexual practices with substance use in complex social environments, presenting significant risks to physical, mental, and sexual health. It is necessary to implement public health interventions to mitigate these risks.

## 1. Introduction

The emerging phenomenon known as chemsex, derived from the combination of “chems” (which was a term widely used in the UK gay community to refer to methamphetamine and GHB/GBL when communicating by phone or SMS at the end of the last century) and “sex”, reflects a practice [1] linked to gay culture in which drugs are used in a sexual context [2]. These substances are used recreationally before or during sexual relations, mostly in private settings (68.4%) and in commercial sex venues such as saunas, sex clubs, or dark rooms in bars (24.5%) with the purpose of enhancing and prolonging the sexual experience [3]. This phenomenon has been declared a public health problem in some places like Madrid or Barcelona with implications for the physical, mental, and sexual health of those who practice it [4,5].

Chemsex should be approached from a holistic perspective, recognizing it as a complex reality that involves distinctive behavioral patterns, underlying challenges, and specific characteristics of the group of gay, bisexual men, and MSM. The reasons motivating drug use in the context of chemsex are diverse, on the one hand, including an increase in excitement and libido, an increase in sexual confidence, a reduction in inhibitions, and an increase in the duration of sexual relations as well as the intensification of emotions [6] to avoid feelings of isolation, shame, stigma, rejection, low self-esteem, and the lack of meaningful connections [7].

The consumption of psychoactive substances can be related to a variety of mental health problems, such as depression, anxiety, psychotic disorders, addictions, and even an increased risk of suicide [8,9,10,11].

Research at the European level has highlighted the diversity in the spread of this phenomenon among different countries and environments, exploring the different substances consumed, as well as the contexts, patterns of use, motivations, and experiences of users [12].

The significant increase in the number of people treated for chemsex addiction in health services between January 2021 and July 2022 is alarming, especially in the community of Madrid. The attention to 351 individuals in this period represents an increase of 702% compared to the 2017–2018 period, when only a total of 50 people were reported [13]. These data reflect a worrying trend that demands immediate action from health authorities and health services. A study developed in Spain reveals that a significant proportion of chemsex participants use multiple substances to enhance the sexual experience, which increases the risk of sexually transmitted infections and mental health problems [14].

Despite the abundance of quantitative studies on chemsex [15,16,17], a growing interest in qualitative research exploring the social and cultural practices associated with chemsex in various countries has recently emerged [4,18] in order to better understand risky sexual behavior, the factors that influence it, and prevention strategies [19,20], as well as the different contexts in which these practices occur [21]. A recent study conducted in Madrid highlights the need to develop a more detailed description [22], which is supported by another article at the national level [11].

Approaches are required that seek to reduce the risks associated with chemsex, contemplating one’s emotional well-being, the responsible management of substance use, and the recovery process. To effectively address the challenges derived from this phenomenon, it is essential to implement a comprehensive intervention strategy that considers its complexity and the individual needs of each user through a multidisciplinary team. This implies understanding the chemsex phenomenon by professionals to identify the individual and contextual complexities that influence this practice. Only by understanding the motivations, social dynamics, and risks involved can professionals design tailored interventions that comprehensively address the needs of users. This understanding not only allows for a more effective response but also promotes an empathetic and prejudice-free approach that fosters trust and multidisciplinary collaboration between healthcare professionals and users, thus facilitating a reduction in the risks and harms associated with this practice.

For all of the above, this study focuses on exploring the perceptions, management, safety, and factors associated with the practice of chemsex.

## 2. Methods

### 2.1. Study Design

This is a qualitative descriptive phenomenological study, based on Husserl’s theoretical framework [23]. Qualitative descriptive designs focus on the words of participants to describe their personal experiences and perspectives on specific phenomena [24]. In particular, descriptive phenomenology maintains a close connection with Husserl’s original conception of phenomenology, centered on creating detailed descriptions of others’ specific experiences [25]. Additionally, this qualitative design allows for the use of a wide variety of data collection instruments and different analytical approaches to describe what is happening and how it is occurring, all from the perspective of the participants [24]. This study was conducted in accordance with the Consolidated Criteria for Reporting Qualitative Studies (COREQs) and the Standards for Reporting Qualitative Research [26]. (See Supplementary File S1).

### 2.2. Experience and Role of Researchers

Ten researchers (7 men and 3 women) participated in this study, of which 3 had experience in qualitative study designs (PdPH, ATR, and EGB), and none were involved in clinical activities. None of the research team members had a prior relationship with the participants. Additionally, 5 researchers had clinical and research experience in the field of mental health (PdPH, JCR, CGN, EVG, and TSS). The data were triangulated with two external researchers (CSO and JMPB). The theoretical framework for this qualitative research, as well as the researchers’ viewpoints and their reasons for conducting this study, were defined before starting this research [27].

### 2.3. Participants

The inclusion criteria were being male, 18 years old, and having reported at least one episode of chemsex behavior during their lifetime. Recruitment was carried out through a purposive sampling process, with the aim of recruiting participants who could respond to the proposed objectives [28]. Potential study participants were contacted through advertisements on social networks and LGBT community organizations. The snowball sampling method was also implemented, asking interviewees to suggest other MSM who were involved in chemsex and might be interested in participating. This strategy facilitated the incorporation of several additional participants into the study, which enriched the sample, reaching data saturation with the fifteenth participant [29]. None of the participants dropped out of this study. Table 1 collects the various demographic data.

### 2.4. Data Collection Instrument

Data were collected between April and June 2024 through in-depth interviews and field notes. These interviews were conducted in a semi-structured manner, following a question guide designed to explore specific topics of interest (Table 2).

The interview guide questions were developed according to the needs of the research team and were agreed upon and verified by the team before starting data collection. Despite the structure, participants were given the freedom to share their experiences without being influenced by the interests of the research team [28]. The interviews were conducted by a researcher with previous experience on the topic and had an average duration of 90 min. They were carried out in person, face to face, at the place and time preferred by the participants. Generally, they chose to conduct them at their residence, in order to maintain a safe environment and preserve their privacy. They were recorded using a voice recorder, transcribed verbatim, and anonymized to safeguard the privacy of the participants. In order to respect privacy and promote greater participation, it was decided not to video record the interviews. Although this choice could imply a loss of information or a methodological bias, it was observed that many participants felt more comfortable and willing to discuss issues related to chemsex and were more inclined to give their consent to participate in the study. Throughout each interview, field notes were taken to provide more detailed information [28]. The transcripts were returned to the participants so they could make additional comments. The data were stored in a secure digital environment, with exclusive access for the research team.

### 2.5. Data Analysis

The data were simultaneously analyzed by a team of three researchers (PdPH, ATR, and EGB), following the methodological approach of the thematic content analysis [30]. This type of analysis, which takes into account the diverse viewpoints of the study participants, is consistent with the proposed design. After collecting all the interviews, an initial coding structure was established based on the concepts and themes of the interview guide. Emerging codes were incorporated through the inductive coding strategy, using active reading. All codings were discussed by the research team until a consensus was reached on the main categories and themes, creating a final matrix (Table 3). The ATLAS-TI- v.24.0 software program was used for the analysis.

### 2.6. Quality and Rigor Criteria

To control the rigor and reliability of the qualitative data, Guba and Lincoln’s criteria were applied [31]. Credibility and confirmability were ensured through a triangulation of the researchers, data collection, and analysis. Team reflection was promoted through meetings and reflective reports. Additionally, participants were offered the opportunity to review the interview recordings, although none made additional comments. To ensure transferability, detailed descriptions of the study were provided, including characteristics of the researchers, participants, contexts, sampling strategies, and data analysis procedures. Confidence in the results was reinforced through an evaluation of the research protocol by an external researcher, focusing on the methods used and the study design.

### 2.7. Ethical Considerations

All participants received information about the study objectives and signed an informed consent form (sent by email) before being interviewed. The interviews were anonymous, voluntary, confidential, and did not collect personal data or devices that could identify the informant. The information was treated confidentially and anonymously by having dissociated data, following the Data Protection Regulation (EU) 2016/679 of the European Parliament and Organic Law 3/2018. The researchers declared no ethical, moral, or legal conflicts. They declared not having received any type of economic compensation. Participants did not receive any type of compensation for responding to the questionnaire. The study was conducted under the Declaration of Helsinki and was approved by the University of La Rioja Committee with verification code (CSV) (D2R1m2Iu3vLVPdIzGZVVnK0h6N558tCyN) for studies in humans through this link: https://sede.unirioja.es/csv/public/index.xhtml;jsessionid=2D8AB94A16FEB14C923EFD5E63C25AF2-n1.ma_07 (accessed on 15 January 2024)

## 3. Results

Fifteen individual interviews were conducted with MSM who identify as cisgender with an average age of 42.1 years. Approximately half of the sample (46.67%) were single, 26.67% in a relationship, 13.33% divorced, and 13.33% married. A total of 80% completed university studies, and 87% are in an active employment situation. In the last month, participants reported having practiced chemsex an average of twice a week. The average time since participants started practicing it is approximately 5.57 years (see Table 1).

### 3.1. Themes

Three thematic blocks with their categories were identified: (T1) contextualization of the practice, (T2) factors associated with the practice of chemsex, and (T3) strategies and interventions for risk and harm reduction (see Table 4).

### 3.2. Contextualization of the Practice

#### 3.2.1. Concept

Chemsex is defined as the practice of maintaining sexual relations under the consumption of psychoactive substances. According to the participants, “*any population can do it, but it is concentrating quite a bit in the homosexual sector*” E13. This activity is not only limited to the sexual act itself but also includes masturbation associated with substance use and a prolongation of the time of sexual relations to intensify the experience. A duality in its perception is reflected: on one hand, as an intense and different experience, “*practicing sex under the consumption of psychoactive substances… is something very fun*” E1 and, on the other, as a potentially problematic activity “*A dark world because it can lead you to drug dependence for feeling that you enjoy or participate in sex and that loses you*” E5.

It was observed that there is no concrete and shared definition of the term “chemsex”. Participants tend not to talk explicitly about the phenomenon, instead using terms such as “chill”, which they unconsciously associate with drug use. Some clearly distinguish between “chill” and “chemsex”, describing the latter as a premeditated meeting to have sex with the use of substances: “*for me chill is not the same as the concept of chemsex, because for me the concept of chemsex that I have in my head was that I am going to meet someone to fuck and in order to do something with him we need to take substances*” E9. In contrast, others see them as similar concepts, suggesting that social interaction in a “chill” can occur naturally in a sexual context, due to the atmosphere created by the substances: “*For me, chill and chemsex are a bit the same… I mean, you are there, you know it is going to happen, that is, there is going to be sexual energy and at the end it triggers them*” E2. As can be seen, “chill” is shown as an activity centered mainly on drug use, although it also includes sex. Some illustrate a second term known as “guarichill” that stands out for its explicit combination of sex and drugs where verbal communication may be limited: “*chill is going to be with colleagues just drinking after a party and getting high and then there is guarrichill which is already a mixture of sex, nobody talks about chemsex*” E6.

#### 3.2.2. Environment

Access is often facilitated through social networks and friendships, “at the beginning through a friend who is more lucid in this world” E8, indicating the importance of personal connections to enter this environment. Another participant adds, “start working the social circles a bit… I mean, you get to Madrid, and there are a lot more chills than you think” E9. This dynamic shows how chemsex is integrated into social life and expands through trusted networks. Dating apps, especially Grindr and Scruff and even others, such as Instagram, are fundamental tools for organizing and accessing sessions, where specific symbols are used: “there is a rocket which generally symbolizes slam and an oak leaf which is for marijuana” E1. These symbols help to quickly identify other interested users facilitating the formation of groups. Although apps are an effective means of contacting people, personal invitations play a crucial role in the integration and participation in these groups.

Initial experiences are often linked to social encounters: “my first experience was already two years ago or more with a guy at home… he came from partying continuously… he told me that he couldn’t stand it and that he was going to shoot up and that if I didn’t want to try it…” E5. This shows how it can start unexpectedly and be related to a search for new sensations after prolonged social events. Also, during the COVID-19 pandemic, the practice of chemsex became popular as a form of fun: “It was during COVID because we were locked up at home… and then I started to know the Chill concept” E1. Reactions to this first experience vary significantly. Some report surprise at the situation: “Innocent of me that we get into a house with everyone in the dark with people fucking around. What’s going on here? Does this exist?” E14. For others, it can be very fun and satisfying: “it was very, very satisfying because I suddenly discovered a very different sex” E15. However, for others, it is something disturbing “the truth is that I was a bit horrified by what I was seeing and I didn’t do anything, however, I stayed a while to observe” E11. This suggests that, although it can be shocking and disturbing, curiosity and observation can play a role in understanding this phenomenon. It also reflects that there is a transition from casual and spontaneous encounters to more organized and structured meetings, reflecting a greater familiarity and comfort with the practice: “at the beginning it was a bit more spontaneous but now it is almost planned or semi-planned” E7.

These practices take place in a variety of places, from apartments to saunas: “*The normal thing is private apartments, you can also do it in saunas*” E10. Saunas can be conducive spaces for the practice of chemsex, providing an intimate and closed environment where they can consume drugs and engage in sexual activities: “*It was when I put these people in the sauna for the first time… we went back to Madrid and it’s been like a whole evolution of mixing substances and partying*…” E9. However, others mention the growing preference for private and controlled environments, such as their own home or that of a close friend: “*People who go out on Fridays to party, since your body is already loaded with drugs, you continue on Saturday in an apartment*” E8.

#### 3.2.3. Motivations

Chemsex provides an intensely pleasurable and satisfying sexual experience compared to traditional ones. It acts as a powerful disinhibitor, allowing shy or introverted individuals to explore without restraint. This liberation facilitates deep emotional connections that might otherwise be limited: “*because it’s the feeling that new people come… that you don’t have limits, that you’re not inhibited and you can do a little bit whatever you want… you know, like another level*” E3. Testimonies reveal how its practice transforms the perception of self and others, offering a space where sexual and emotional expression reaches intensified levels. It represents a way to connect emotionally, overcome personal barriers and enjoy a sense of belonging and acceptance: “*I have a great time, I’m just as sociable, I’m just as awake… I’m not drunk, I don’t have memory lapses*” E9. For many, it represents an opportunity to confront and overcome personal insecurities, including internalized homophobia. Drug use in this context provides an avenue to evade self-judgment and achieve greater self-acceptance. This is evidenced by how some individuals find, in chemsex, an informal therapeutic tool to improve their self-esteem and confront past traumas: “*To avoid myself, to stop judging myself… I feel better about myself, more self-esteem*” E5.

### 3.3. Factors Associated with Chemsex

#### 3.3.1. Physical Health

Experiences reveal that chemsex can have significant physical effects, highlighting prolonged exhaustion and other adverse symptoms: “*I am now exhausted…Not only does it induce considerable physical exhaustion, but it can also compromise the immune system, causing a decrease in defenses resulting in recurrent symptoms such as shingles or colds*”. “*Yes it’s true that my defenses go down, I always get shingles or you’re with a cold or whatever*” E13. These effects are directly related to the duration in days of the practice and the consumption of drugs: “*It depends on the number of hours you have been there, the longer it goes on, the more wasted you are the next day*” E10.

#### 3.3.2. Sexuality

Prolonged substance use during sessions can result in a number of sexual health problems. Difficulties with erections and ejaculation problems have been reported: “*there are times when if you don’t get into that mental sexual suggestion, you won’t get hard… it will work to get you hard but it’s not going to make you horny per se*” E9. This negatively affects sexual function and the body’s physical response: “*you’re not able to get horny and it’s like… no, like, yes, I can, I want to get that libido back and what made me horny in the first place*” E9. Drug dependence is also a concern for some, who feel that they need substances in order to enjoy sex, which can impact both their physical health and their long-term mental well-being: “*I don’t have a serious problem, but it worries me knowing that every time I’m going to have sex, I have to pull drugs and well, I’m a little worried about dependence in general*” E8. In the short term, negative effects include immediate sexual problems, but in the long term, it can have even more profound impacts related to persistent erectile function difficulties, concentration problems, and other adverse health effects: “*The days after, concentration and that kind of thing very bad. Erections type also fatal*” E11.

#### 3.3.3. Social Impact

These changes can affect personal relationships by complicating the individual’s social and professional dynamics. It can result in a significant distancing from individual and social responsibilities: “*It has cost me a divorce… I have had to stop working because I could not under these conditions…*” E9. Frequent cancellations of plans and social commitments due to chemsex were mentioned as a negative consequence: “*Negative part obviously are all those feelings you have afterwards… that of canceling plans, to be failing your friends*” E9. These problems include noncompliance with work and domestic obligations, as well as the avoidance of social and family commitments, contributing to the overall deterioration of quality of life and interpersonal relationships.

#### 3.3.4. Economic Expenditure

Chemsex can involve significant expenses, with costs that vary widely according to various factors: “*like really crazy I could have spent… getting high all night 100 euros*” E3. Even higher figures are mentioned, presenting a considerable expense with a potential negative impact on the personal economy: “*500 euros is a considerable expense that affects your economy, and it is something you should take into account*.” E2. These data underline that this practice not only affects one’s health but also their financial stability, highlighting the need for the responsible management of expenses associated with these practices.

#### 3.3.5. Emotional Impact

It is reflected that chemsex can have significant impacts on the mental health of those who participate, manifesting itself in various forms of emotional distress and profoundly affecting the quality of life of the individuals involved. For many, it entails intense feelings of guilt and remorse: “*Beyond the physical deterioration, the economic expense and an initial distancing from my family and friends… that I managed to stop in time, it has not gone any further but it could have gone much further*” E5.

### 3.4. Risk and Damage Reduction Strategies and Interventions

#### 3.4.1. Seeking Emotional Support

Participants expressed the need for the support of professionals to provide them with emotional support to be able to cope with this situation: “*I think talking, being open about it…. generally for me, the more you talk about it, the easier it is to control it*” E3. However, access to these support services is not easy or does not meet their expectations: “*there is a waiting list where you go to get help and to be seen by your first therapist it’s a month and a half*” E6. Furthermore, when you arrive there, you do not have access to the psychologist or psychiatrist or social worker or group therapies, and when patients do receive help, their experience is not always satisfactory: “*I tell you with this professional who is a little bit rude who treated me… I felt very judged by her*” E9.

People who practice chemsex consider that it is also necessary to make this phenomenon, recognized as a public health problem, more visible: “*I don’t think that chemsex is being dealt with in a good way because it is starting to be talked about and verbalized because it is a reality that has been thrown at us, but when you go to seek help there is not a single resource that can help you.*” E6. They have observed the work of groups, associations, and medical centers but indicate the absence of general campaigns by the government: “*I have seen groups and associations and medical centers that do the work but I have not seen a government that has made a campaign at the general level*” E9.

#### 3.4.2. Prevention and Health Promotion

The perception among those who practice chemsex is that, in addition to the lack of professional support, it is crucial to support them with educational initiatives similar to those implemented in other European Union countries: “*look I have seen it in other cities like Berlin or Paris, but here in Spain I sincerely wonder why there is not more… there really is not much information about it… neither in the specialized media aimed at the gay community nor have I seen much presence of leaflets in places of slutting or saunas or dark rooms*” E8. They identify specific training areas: “*Well, especially with information about the type of drugs that are consumed, information about the immediate and secondary effects. And then also a topic of sex education, because as I told you, condoms are not used in the chills*.” E7. They also propose improving access to tools that can help them reduce risks in substance use: “*I had talked about Energy Control… a place where they analyze substances… if that kind of service were more accessible that would probably be a good action to really know what you are getting into, maybe people would see the shit they sell you*…” E1. Given the widespread use of PrEP, they also consider expanding information about this treatment: “*PrEP is being given to everyone in a very general way, without informing, without explaining, without saying that the diseases are still there, and that no matter how much you take it, not only will you catch an STD, but anything can happen to you*” E5.

**Figure 1:**
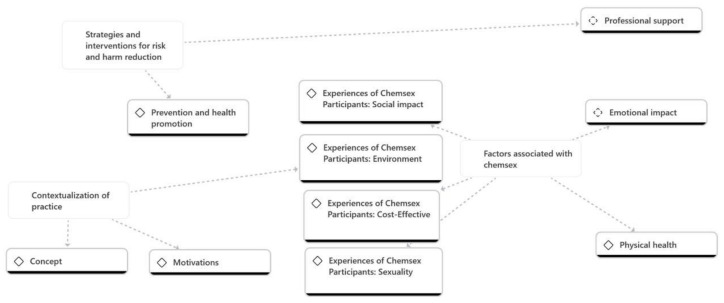
Qualitative data analysis.

These findings illustrate the complexity of the intervention, highlighting the importance of effective strategies and the consideration of various alternatives to address simulated mental health situations. Figure 1 allows us to establish the following themes: 1. Contextualization of the practice (Concept, Environment, Motivations). The second theme deals with the factors associated with chemsex (Physical health, Sexuality, Social impact, Economic impact, Emotional impact). The third theme deals with risk and harm reduction strategies and interventions (Seeking professional support, Prevention and health promotion). All of them are interrelated with each other and with the chemsex phenomenon through the ATLAS-Ti program.

## 4. Discussion

This study offers a deep look into the contextualization, perceptions, and social environments of chemsex, highlighting its complexity and the multiple dimensions that characterize it. This analysis provides essential information for a greater understanding of the phenomenon. A complex intersection between sexual practices, substance use, and social contexts is revealed [21]. Participants describe it as the use of psychoactive drugs during sexual activities, affecting not only physical aspects but also significant emotional and social dimensions [32]. Other studies have agreed on a definition that links the consumption of illicit drugs to enhance and extend sexual activity [20,33] and achieve greater intimacy [34].

Initial experiences often involve prolonged social encounters, characterized by a variety in the frequency and duration of sessions, where participants share and take advantage of substances [35]. Access to these practices is facilitated through social networks and personal contacts through the use of geolocation applications to plan sessions. In addition, various motivations for engaging in chemsex are identified, such as curiosity, disinhibition, an increased libido and sexual pleasure, increased confidence, and an intensification of sensations. This framework comprises how social aspects, substance availability, and individual motivations intertwine to shape the dynamics of participation in chemsex [9,36]. It is not only presented as a hedonistic practice; it is revealed as a means to satisfy both deep physical and emotional needs [37]. This phenomenon facilitates an intensified exploration in both the sexual and emotional realms and serves as a means to overcome personal and social barriers [38]. For many participants, it represents a significant way to establish emotional connections that promote a sense of well-being and acceptance, aspects that are often difficult to find in other areas of their lives [35]. Although it can provide intense satisfaction in the moment, chemsex poses serious long-term health concerns. From sexual health problems to an alarming increase in mental health issues, these practices can have lasting consequences that affect the physical, social, and mental well-being of the individuals involved. In line with our study, a recent study reflects that it not only deteriorates physical health but also significantly aggravates emotional problems, such as guilt, frustration, internalized homophobia [17], and psychological difficulties, even increasing the risk of suicidal thoughts in some cases [39].

There is a clear demand for professional emotional support, underlining difficulties such as long waiting lists and negative experiences with some therapists. Interactions with health professionals and access to harm reduction information are limited in this population, despite the high risk and potential significant adverse health outcomes. The results indicate the need for innovative and combined approaches to reach this population, including peer support networks, anonymous counseling, and changes in community attitudes towards chemsex [40]. Furthermore, the importance of interventions being accessible, personalized, and non-judgmental to provide adequate care and effective support was emphasized [11]. They criticize the lack of government campaigns and specific resources, despite the existence of initiatives from collectives and medical centers. By understanding the perceptions and experiences of these individuals, nurses can offer more comprehensive and personalized support, thus improving the quality of care and promoting a safer and more understanding health environment, allowing for the development of more effective and empathetic care strategies for users who participate in these practices, addressing both their physical and mental health needs. In addition, they highlighted the urgent need for educational initiatives similar to those in other European countries and for greater visibility of the issue in the media and places frequented by the gay community. For all of the above, it is interesting to make these public health problems visible, so that risk and harm prevention strategies can be implemented to promote the integral health of those participating in them [41].

Recognizing the possible influence of social desirability biases on certain participants is crucial. Additionally, the topics covered in the interviews, such as sexual behavior and illicit drug use, could have influenced the responses of some participants, leading them to alter them to present a more favorable image of themselves. Lastly, it is important to mention that the sample was composed exclusively of men residing in Spain, most of whom had a high educational level. Although this demographic group is relevant, given the importance of the country as a gay tourism destination, these characteristics limit the generalization of the results. Despite these limitations, this study managed to compile a significant variety of experiences related to chemsex, providing a detailed view of the phenomenon.

In discussing our study, it is important to consider that the sample was predominantly composed of individuals from a specific geographic area, which may have influenced the socioeconomic profile of the participants. This geographic particularity may partly explain the high educational and acquisitive level observed in our sample, as the socioeconomic factors of the region may not be representative of the general population or the broader group of MSM. However, it is relevant to note that similar findings have been documented in quantitative studies at the national level [11], where a trend toward higher educational and acquisitive levels among participants has also been recorded. This suggests that the observed phenomenon is not unique to the region studied but may reflect a broader trend in the general population. Despite these observations, it is important to recognize our approach’s limitations and consider that future research with more diverse samples could provide a complete and more representative picture of the chemsex phenomenon in different socioeconomic contexts.

Future research with a more diverse sample could provide a broader understanding of the chemsex phenomenon across different socioeconomic groups.

### Nursing Implications

Chemsex presents various risks to the physical, psychological, and social health of participants, highlighting the need for harm reduction interventions. Key strategies include education on safe substance use; access to risk reduction materials, such as condoms and clean needles; needle exchange programs; and psychological support services. These nursing interventions seek to minimize the adverse consequences of chemsex, such as sexually transmitted infections and mental health problems.

To design effective interventions, it is crucial to understand the perceptions and motivations of users. This can be achieved through qualitative research and community participation, ensuring a non-judgmental approach that fosters trust and acceptance of interventions. Nursing professionals play a vital role in this context, conducting health assessments, providing education on safe practices, offering psychological support, and referring users to specialized services. Their participation is essential for the implementation of harm reduction strategies that promote the integral well-being of chemsex participants.

## 5. Conclusions

Chemsex is a growing and continuously evolving phenomenon whose definition is not yet clearly established. It reveals a complex interaction between sexual practices, substance use, and social environments, with multiple associated motivations and risks. Although it can provide an intense and different experience, it also presents serious long-term health concerns, including various dimensions: physical, social, sexual, and mental. It is crucial to design risk reduction and public health strategies that address these dimensions to promote comprehensive well-being among participants.

## Figures and Tables

**Table 1:** Participant characteristics (n = 15).

Participant	Age	Employment Status	Educational Attainment	Relationship Status	Type of Relationship	Time since Starting to Practice Chemsex	Frequency of Chemsex Practice
E 1	43	Employed—public sector	University degree or higher	Single	-	4 years ago	Two to three times a month
E 2	48	Employed—public sector	University degree or higher	Single	-	5 years ago	Once a month
E 3	47	Employed—private sector	University degree or higher	Single	-	5–6 years ago	Once or twice a month
E 4	48	Employed—public sector	University degree or higher	Single	-	11 years ago	Once a month
E 5	44	Employed—private sector	University degree or higher	Single	-	1 year ago	Four times a month
E 6	43	Unemployed	University degree or higher	Divorced	-	9 years ago	Twice a month
E 7	38	Employed—private sector	University degree or higher	Single	-	1.5–2 years ago	Twice a month
E 8	54	Employed—private sector	University degree or higher	Divorced	-	4 years ago	Twice a month
E 9	30	Employed—public sector	University degree or higher	Couple	OR	2 years ago	Twice a month
E 10	33	Unemployed	University degree or higher	Couple	OR	2.5–3 years ago	Once a month
E 11	41	Employed—private sector	Baccalaureate	Married	OR	10 years ago	Twice a year
E 12	56	Employed—private sector	University degree or higher	Married	OR	21 years ago	Once every Three months
E 13	34	Employed—public sector	University degree or higher	Couple	OR	3 years ago	Twice a month
E 14	30	Employed—public sector	Baccalaureate	Couple	OR	2 years ago	Once a month
E 15	43	Employed—private sector	Baccalaureate	Single	-	1.5 years ago	Twice a month

E: interview; OR: open relationship.

**Table 2:** Semi-structured question script.

What does chemsex mean to you? How long ago did you start having sex under the influence of drugs, and since then have your relationships always been associated with drug use? Do you worry about your drug use, and why? What would you say is the reason/detonator for you to practice chemsex? Where does it usually take place and what is the access like? What moments, situations or factors do you think favor it the most, and which ones make it more difficult? In the days before and after chemsex, how do you feel? How was your first experience? How has the practice of chemsex affected you in your life? Do you feel that you have enough information to manage pleasures and reduce risks in the practice of chemsex? What changes have you noticed in your mental health since practicing chemsex? What harm and risk reduction interventions do you know about from public entities?

**Table 3:** Analysis procedure.

Analysis Phases	Process/Actions
Familiarization with the data	- Transcription of the data - Examine and review text content - Identify initial concepts
Coding	- Systematically classify relevant fragments of the text - Verify the relevant contents of each code
Categorization	- Group the classifications by similarities - Check consistency between categories and assigned classifications, as well as with all data - Create a map of the categories - Define and name the categories
Selecting significant extracts as examples	Relate the findings to the research question to prepare the final report

**Table 4:** Themes and categories.

	Themes (T)	Categories
**T1**	Contextualization of the practice	Concept Environment Motivations
**T2**	Factors associated with chemsex	Physical health Sexuality Social impact Economic impact Emotional impact
**T3**	Strategies and interventions for risk and harm reduction	Seeking professional support Prevention and health promotion

## Data Availability

The data are available upon request of the first author.

## Supplementary Materials

The following supporting information can be downloaded at: https://www.mdpi.com/article/10.3390/nursrep14030172/s1, File S1: COREQ checklist.

## Public Involvement Statement

There was no public involvement in any aspect of this research.

## Guidelines and Standards Statement

This manuscript was written following the COREQ guideline for qualitative research.
